# Good Laboratory Practices and Safety Assessments: Another View

**DOI:** 10.1289/ehp.0901755

**Published:** 2010-05

**Authors:** Tony Tweedale

**Affiliations:** R.I.S.K. Consultancy (Rebutting Industry Science with Knowledge), Edinburgh, Scotland, E-mail: tony.tweedale@phonecoop.coop

In a letter responding to an article by Myers et al. (2009), Becker et al. (2009) claimed that industry’s Good Laboratory Practices (GLP)-compliant studies are superior to traditional academic peer-review in predicting the risk of toxic agents. I have read almost 30,000 experimental, etiologic, and epidemiologic papers (most in part), and it is evident that industry GLP studies do not report the same risks of a chemical when published in peer-reviewed studies from academia. This may be explained by biases in industry experiments and epidemiology, especially in design, due to the financial interests of industry sponsors—some receiving billions of dollars in revenue per chemical each year. For pharmaceuticals, dozens of published reviews show a strong correlation between industry sponsorship and findings of safety; I know of four such strong correlations in studies of industrial chemical risks (Bekelman et al. 2003; Fagin and Lavelle 1999; Swaen and Meijers 1988; vom Saal and Hughes 2005).

Becker et al. (2009) relied on a commentary by a former editor at the *Nature* research journals (Jennings 2006) to claim that peer-review gives inferior data compared with GLP studies. Actually, Jennings (2006) wrote about improving, not abandoning, peer review. He presented data showing that the long-term value of scientific papers in neuroscience (judged by experts) correlates with the quality of the journals in which they were published (based on impact factor). That is a cardinal finding because industry supports various journals and their scientific associations, but their GLP studies are rarely published in high-quality journals (again, based on my readings). Evidently, industry’s GLP data are not reliable enough to publish, while financial independence of authors and editors, as well as peer review, are markers of good quality data.

Since the widespread experimental testing frauds at Industrial Bio-Test Laboratories (Schneider 1983) and Craven Laboratories (U.S. Environmental Protection Agency 1994), which generated the GLP reforms, industry has issued oceans of GLP-compliant studies for submission to regulatory agencies. Few are submitted for publication, but almost all (in my experience) are submitted to journals that publish many industry-sponsored studies.

Critically, industry and their regulatory agencies took the opportunity proferred by the requirement to comply with GLP to exclude almost all academic high-quality, non-GLP studies from risk assessments of existing chemicals (and the toxicity of new agents are primarily evaluated by the parties who want to sell it). For existing chemicals, I have always found that the effective toxicity doses in regulatory (GLP) studies are higher than those in the peer-reviewed literature, for several end points.

It is important for individuals who value the contributions that science makes to society (reliable data)—or those who are cautious about toxicity of low-dose and cocktail agents that may affect biochemical signals, especially during development—to continue lobbying public agencies to incorporate academia’s peer-reviewed studies and to use disclosure of financial interests to give appropriate credence to industry’s data in chemical risk assessments. I also call on independent academics to be less competitive and make their methods and data more freely available.
